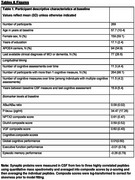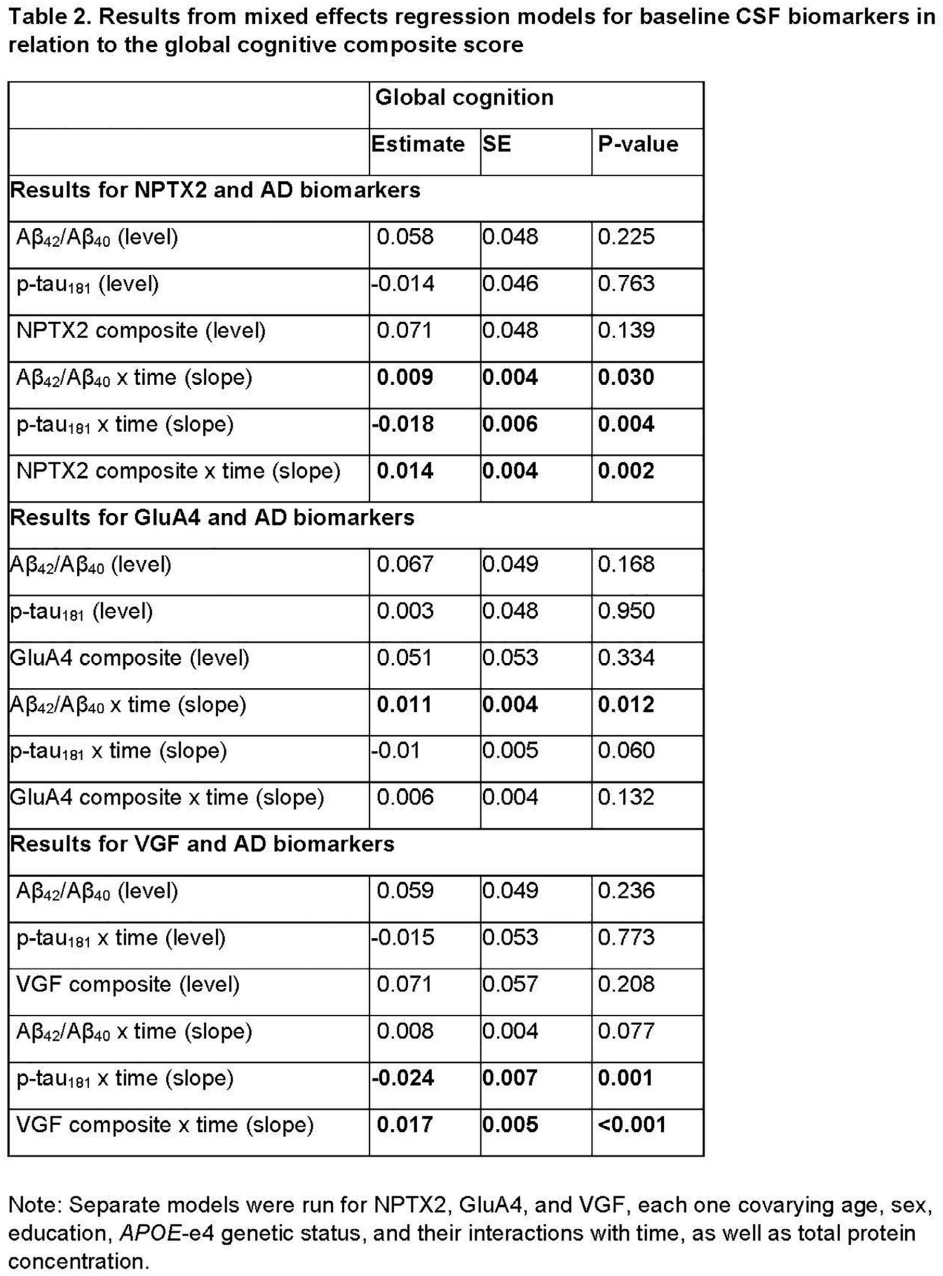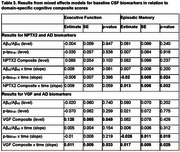# CSF synaptic markers are associated with cognitive resilience in aging and preclinical Alzheimer's disease

**DOI:** 10.1002/alz70856_103160

**Published:** 2025-12-24

**Authors:** Claire L Anderson, Alden L. Gross, Corinne Pettigrew, Juan Vazquez, Hang Wang, Abhay Moghekar, Sungtaek Oh, Chan‐Hyun Na, Marilyn S. S. Albert, Paul F Worley, Anja Soldan

**Affiliations:** ^1^ Johns Hopkins University School of Medicine, Baltimore, MD, USA; ^2^ Center on Aging and Health, Johns Hopkins University, Baltimore, MD, USA; ^3^ Johns Hopkins Bloomberg School of Public Health, Baltimore, MD, USA

## Abstract

**Background:**

Recent evidence suggests that higher levels of the synaptic marker neuronal pentraxin 2 (NPTX2) and synaptic neurosecretory protein VGF (also known as secretogranin VII) may confer resilience to Alzheimer's disease (AD) pathology and other neurodegenerative diseases. Because little is known about these and related synaptic proteins during the preclinical phase of AD, this study examined the association between baseline levels of VGF, NPTX2, and the associated AMPA glutamate receptor subunit 4 (GluA4), measured in cerebrospinal fluid (CSF), with long‐term trajectories in global and domain‐specific cognitive performance among individuals who were cognitively unimpaired at baseline.

**Methods:**

Baseline CSF levels of NPTX2, GluA4, and VGF were measured using parallel reaction monitoring mass spectroscopy from 269 cognitively unimpaired BIOCARD participants (*M* baseline age=57.7 years, mean cognitive follow‐up=15.9 years; see Table 1). Baseline CSF levels of Ab_1‐42_/Ab_1‐40_ and *p*‐tau_181_, biomarkers of AD pathology, were measured using the Fujirebio Lumipulse G1200 assays. Linear mixed effects models evaluated associations between baseline NPTX2, GluA4, and VGF with level and rate of change in a global cognitive composite score. If significant, follow‐up models examined associations with domain‐specific executive function and episodic memory composite scores.

**Results:**

More abnormal AD biomarker levels were associated with greater declines in the global cognitive composite score. After accounting for AD biomarker levels, higher NPTX2 and VGF levels were associated with less decline in the global (both *p*≤0.002; Table 2) and episodic memory (both *p* <0.03; Table 3) scores. Higher VGF was additionally associated with less executive function decline (*p* = 0.03; Table 3), with similar patterns for NPTX2 (*p* = 0.06). GluA4 was not significantly associated with level or change in any of the cognitive composite scores (all *p*>0.08).

**Conclusion:**

Baseline levels of synaptic proteins NPTX2 and VGF were associated with reduced rates of cognitive decline, independent of AD biomarker levels. The results are consistent with the hypothesis that these markers may impart resilience to cognitive decline in the presence of AD pathology and may hold promise as potential therapeutic targets for AD.